# Time-Series-Based Personalized Lane-Changing Decision-Making Model

**DOI:** 10.3390/s22176659

**Published:** 2022-09-02

**Authors:** Ming Ye, Lei Pu, Pan Li, Xiangwei Lu, Yonggang Liu

**Affiliations:** 1 Key Laboratory of Advanced Manufacturing Technology for Automobile Parts, Ministry of Education, Chongqing University of Technology, Chongqing 400054, China; 2 State Key Laboratory of Mechanical Transmissions, College of Mechanical and Vehicle Engineering, Chonqing University, Chongqing 400044, China

**Keywords:** autonomous vehicles, lane-change decision, driving style, LSTM, interaction

## Abstract

In recent years, autonomous driving technology has been changing from “human adapting to vehicle” to “vehicle adapting to human”. To improve the adaptability of autonomous driving systems to human drivers, a time-series-based personalized lane change decision (LCD) model is proposed. Firstly, according to the characteristics of the subject vehicle (SV) with respect to speed, acceleration and headway, an unsupervised clustering algorithm, namely, a Gaussian mixture model (GMM), is used to identify its three different driving styles. Secondly, considering the interaction between the SV and the surrounding vehicles, the lane change (LC) gain value is produced by developing a gain function to characterize their interaction. On the basis of the recognition of the driving style, this gain value and LC feature parameters are employed as model inputs to develop a personalized LCD model on the basis of a long short-term memory (LSTM) recurrent neural network model (RNN). The proposed method is tested using the US Open Driving Dataset NGSIM. The results show that the accuracy, F1 score, and macro-average area under the curve (macro-AUC) value of the proposed method for LC behavior prediction are 0.965, 0.951 and 0.983, respectively, and the performance is significantly better than that of other mainstream models. At the same time, the method is able to capture the LCD behavior of different human drivers, enabling personalized driving.

## 1. Introduction

Autonomous driving is expected to help reduce traffic accidents, reduce the workload of drivers, and improve the quality of transportation, which has become a research hotspot in recent years [1]. However, the majority of people are skeptical regarding self-driving cars. A poll conducted in Germany found that roughly 25% of respondents were hesitant to utilize self-driving cars because their set driving patterns made them feel psychologically constrained and uneasy [2]. According to studies, in addition to legal and safety considerations, fulfilling user expectations, user acceptability, and accessibility to functional implementation are the fundamental requirements for the effective deployment of autonomous cars [3]. Therefore, enabling autonomous vehicles to have more accurate, comfortable, and personalized behavioral decisions is the key to improving autonomous driving technology.

The LCD problem is one of the more complicated and demanding challenges in autonomous driving technology, and has been intensively investigated by researchers at home and abroad [4]. Existing LCD methods can roughly be divided into two categories: rule-based methods and learning-based methods. Rule-based decision-making methods plan the behavior of autonomous vehicles, and establish a decision-making behavior library based on driving rules, knowledge, experience, and traffic rules [5]. The Gipps [6] model and the CORSIM [7] model are examples of traditional rule-based LCD models. Finite state machines have also frequently been employed in autonomous driving LCD systems, providing an example of rule-based decision-making techniques. Wang et al. [8] proposed a driving LCD model based on the finite state machine, which output the best driving behavior in the current scene by means of a benefit evaluation model. Qi et al. [9] designed a hierarchical finite state machine behavioral decision-making model. Rule-based decision-making methods can handle the general driving environment well, but they are designed in a fixed way, and their mode cannot be adjusted for drivers with different driving styles.

The monitors and sensors of autonomous cars are now able to record the motion state parameters of both the vehicle in front and other nearby vehicles thanks to advancements in autonomous driving decision-making technology. As a result, it is possible to study how vehicles make decisions by mining historical vehicle motion data [10], which is one of the key causes for the progressive rise in popularity of learning-based methodologies. Liu et al. [11] built an autonomous LCD model based on benefit, safety, and tolerance using a Bayesian parameter-optimized support vector machine method. Ma et al. [12] constructed a driving agent model based on a Bayesian network, which integrated vision and decision making. Tang et al. [13] proposed a lane change prediction method based on an adaptive fuzzy neural network. Xie et al. [14] utilized random forests to simulate LC maneuvers from the standpoint of traffic incidents. Díaz-Álvarez et al. [15] employed artificial neural networks to model the behavior of drivers performing LC tasks. Human driving behaviors are typically extremely nonlinear and complicated movements that are challenging to adequately simulate using traditional shallow machine learning models.

Deep neural networks (DNNs) are able to mimic discretionary lane changing (DLC) operations on roads more accurately, and may implicitly include memory effects in their structures [16]. DNNs have been shown to possess excellent application potential for behavioral decision making in complicated settings, such as urban roadways and crossroads [17]. On the basis of NGSIM data, Zhang et al. [18] established a vehicle following and LC simulation model using an LSTM model optimized by the Hybrid Retraining Constraint (HRC) training method. Zhang et al. [19] utilized XGBoost to construct an LC prediction model on the basis of the NGSIM dataset with selected high-dimensional driving feature data. Fang [20] used a deep belief network (DBN) to build an LCD model, and trained the network using NGSIM data, and the results showed that the network outperformed the BP neural network. Although these methods can achieve good LC predictions in driving scenarios, they have limitations with respect to modeling interactions with each other.

The analysis of the above decision-making approaches reveals that less study has been performed on personalized behavioral decision making and more emphasis has been placed on security and scene coverage in current LCD methods. At present, most of the personalized driving research focuses on the design of assisted driving systems and the identification of driving style. In fact, different drivers show different personal preferences in terms of risk perception, ride comfort and travel efficiency. Therefore, from a driving style perspective, enabling autonomous driving to capture human decision-making behavior is expected to provide drivers and occupants with personalized choices. In addition, the above methods only consider the feature quantities (e.g., speed, acceleration and distance) related to the motion state of the subject vehicle when modeling behavioral decisions. However, when an autonomous vehicle is operating in the driving environment, it forms a whole in which both it and the surrounding vehicles affect each other. Autonomous vehicles cannot make precise behavioral decisions by only considering the characteristics of the motion state of the subject vehicle.

To address these issues, on the basis of the LSTM recurrent neural network model, this paper establishes an LCD model that considers the driving style of the SV and includes interactions. Firstly, personalization factors (driving characteristics and driving style) are introduced into the algorithm model, enabling autonomous vehicles to capture individual characteristics and human decision making, with the aim of achieving personalized driving, while also improving the adaptability of automated driving systems to human drivers. Then, the interaction between the autonomous vehicle and surrounding vehicles is modeled by constructing a gain function to improve the accuracy of the decision-making method. At the same time, the driving environment is extended to three lanes, with the autonomous vehicle being able to perform lane keeping and left–right LC, which is more general. The contributions of this study can be summarized as follows:

(1) A driving style recognition method for autonomous vehicles on highways is proposed, and characteristic variables such as speed, acceleration and headway are selected for quantitative analysis.

(2) The interaction between the main vehicle and the surrounding vehicles is modeled through the gain function, which enhances the understanding of the scenario by the autonomous vehicle.

(3) A personalized decision-making model with interaction is established. The behavioral feature data and LC gain values of different driving styles are used as the input of the LSTM model, and then the time series relationship of various states in the process of lane change is learned.

The rest of this paper is organized as follows: Section 2 briefly describes the overall framework and data processing modules of the paper; Section 3 describes the scheme for driving style identification; Section 4 constructs a personalized LCD model; Section 5 describes the model evaluation and analysis of the results; and Section 6 presents the conclusions.

## 2. The High-Level Framework of the Personalized LCD Model

This section introduces the high-level framework of an LSTM-based personalized LCD model and proposes a scheme for NGSIM data processing. The following content will be elaborated on the basis of this framework.

### 2.1. The High-Level Framework

**Figure 1:**
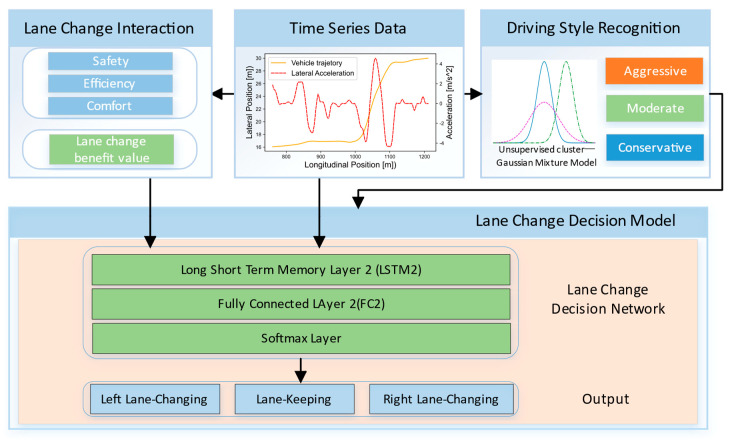
Illustration of the high−level framework for the personalized LCD model.

The high-level architecture of the personalized LCD model proposed in this paper is shown in Figure 1, and includes four modules: data processing module, driving style recognition module, LC interaction module, and LCD module. Among them, the main function of the data processing module is to extract the state feature information between the SV and the surrounding vehicles. The information of the SV will be used to learn the driving style by means of the unsupervised clustering algorithm GMM. GMM is able to obtain the best cluster labels for each vehicle on the basis of handcrafted features. The LC interaction module is mainly used to model the interaction between the SV and the surrounding vehicles. Here, the gain function is used to describe its interaction, and the left LC gain value, lane keeping gain value, and right LC gain value of the SV are obtained. On the basis of driving style recognition, the LC feature parameters and LC income value are used as the input of the decision model, and then the LCD command is obtained.

### 2.2. NGSIM Data Processing

The NGSIM dataset provides actual driving trajectory data on US roadways. According to the study demands, the data of the I−80 and US−101 expressway portions were selected as the original data to develop the LC behavior sample library. The NGSIM information is gathered from various time periods for different places, and it is able to reflect congestion and moderate traffic conditions. A key concern with raw NGSIM data is that there are several substantial errors in the data, such as irregular and missing values for vehicle speed and acceleration. Significant outliers that are 20 times greater than the mean are termed outliers, and outliers are replaced by the mean. Then, the log−exponentially weighted moving average filtering technique (sEMA) introduced by Thiemann [21] is used to filter and preprocess the NGSIM data.

The NGSIM data provide the trajectory data of all cars on the road, including the speed, acceleration and location information of each vehicle every 0.1 s. It includes three vehicle types: motorbikes, cars and trucks. This research focuses solely on the trajectory data of cars. Here, we conduct the following data filtering:

- Eliminate the vehicle data without the preceding vehicle. Such data will lead to missing parameters for driving style clustering and LCD.
- Considering that there may be MLC vehicles in the auxiliary lanes (lanes 1 and 6), extract the relevant data of the middle lanes (lanes 2, 3, 4 and 5).
- Select the vehicle data of the 10 s complete LC trajectory, that is, 50 frames of data (5 s) before the lane crossing and 50 frames of data (5 s) after the crossing.
- Remove continuous lane change and non-adjacent LC behavior data.

After performing the data processing described above, 403 left LC samples, 121 right LC samples and 1400 lane keeping samples are extracted from the NGSIM dataset. Considering that there are fewer LC samples, this will affect the training accuracy of the decision model. Therefore, data augmentation is performed for the LC samples using a random sampling approach employing a random sampling rate between 0.8 and 0.9 per iteration. For each sample sequence, the sampling unit randomly creates a number within the range of the sampling rate and randomly extracts the data to construct a subsequence of the main sequence. Finally, 1208 left LC samples, 363 right LC samples and 1400 lane keeping samples are obtained, for a total of 2971 samples.

## 3. Driving Style Recognition Based on GMM

In this section, the Gaussian mixture model (GMM) is used to generate a unique driving style for each vehicle. The Gaussian mixture model is an extension of the Gaussian model, and it is also a linear combination of multiple Gaussian distribution functions. GMM is the fastest−learning probability model. Its principle is to construct the most suitable mixed multi-dimensional Gaussian distribution model by fitting the input dataset [22]. The Gaussian mixture model GMM can be described as follows:

(1) $$p\left(x_{i}|\theta \right)=\overset{k}{\underset{n=1}{\sum }}\pi_{k}N\left(x_{i}|\mu_{k},\Sigma_{k}\right)$$

Here, $x_{i}$ denotes multidimensional feature data. *θ* is the parameter of the Gaussian mixture model, which can be expressed as $\theta =\left\{\pi_{k},\mu_{k},\Sigma_{k}\right\}$. $\pi_{k}$ is the weight of each Gaussian distribution, meaning the probability of each cluster class being selected, and $\sum_{k=1}^{K}\pi_{k}=1$, where μ and *Σ* are the mean and covariance parameters of the multivariate Gaussian function, and K is the number of models. $N\left(x_{i}|\mu_{k},\Sigma_{k}\right)$ is the univariate Gaussian distribution function in this case, and its form is as follows, where $\Sigma_{k}=\sigma_{k}^{2}$, $\sigma_{k}$ represents the standard deviation of the kth class:

(2) N(x|μ,∑)=1(2π)D2|Σ|12exp[−12(x−μ)TΣ−1(x−μ)]

When clustering datasets, the Gaussian mixture model for unknown parameters is not able to determine which potential components each data point originates from, so it is necessary to estimate the parameters of the Gaussian mixture model. The expectation maximization algorithm (EM) is a commonly used algorithm for GMM parameter estimation. EM is a maximum likelihood estimation algorithm that iteratively computes the maximum value of the cost function [23].

(1) Selection of the Number of Clusters: GMM−based clustering requires the number of clusters *k* to be pre−specified, and finding the optimal value of *k* is a challenge. To obtain the optimal number of clusters, this paper adopts the Akaike Information Criterion (*AIC*) and Bayesian Information Criterion (*BIC*) to evaluate the performance of GMM clustering, where *k* is the number of clusters, *N* is the number of data points, and *L* is the maximum likelihood of the objective function. The formulas for calculating *AIC* and *BIC* are as follows:

(3) AIC=2k−2ln(L)

(4) BIC=kln(N)−2ln(L)

(2) Feature Selection: Different drivers have distinct driving styles when they drive. This paper classifies drivers by analyzing the identification parameters of each driver’s driving characteristics. There is no uniform standard for the selection of driving style characterization parameters, and different scholars choose different indicators. The driving style index parameters selected in this paper are shown in Table 1, in order to fully characterize the driving style of the driver.

In this research, the influence of GMM on driving style recognition is investigated. The GMM−based driving style identification algorithm is able to determine the driving style of the SV on the basis of few driving behavior data. From Table 1, it can be seen that feature information such as speed, acceleration, lateral speed, and spatial headway time distance of the SV are extracted. At the same time, statistics such as standard deviation, mean and maximum values are introduced to strengthen the robustness of the GMM clustering method recognition.

Here, it is assumed that the driving style of each vehicle does not change at a particular time. The data for the statistical characteristics of the three SV driving styles are shown in Table 2. The driving styles can be defined as aggressive, moderate, and conservative on the basis of the characteristic quantities of lateral speed maximum, acceleration mean, and headway time distance. The definition of these three groups of names is only a reflection of the clustering of the SV driving styles and does not affect the further analysis of personalized LCD.

**Figure 2:**
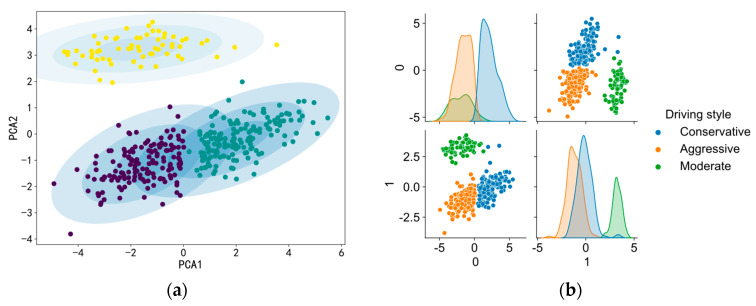
Cluster visualization for 3 driving styles. (**a**) Distribution of three driving styles with respect to two principal components. (**b**) Clustering results based on principal components.

Using the built GMM clustering algorithm, 481 driving behaviors were classed as aggressive, 1286 were classed as moderate, and the remainder (1204) were classed as conservative. It can be seen from Table 2 that drivers with different driving styles exhibit significantly different driving behaviors. Aggressive drivers tend to drive at faster speeds, with shorter following distance and smaller headway. However, conservative drivers chose lower speeds, longer following distances and larger headway distances in order to drive carefully. Figure 2 presents a visualization of the three clusters of driving styles. As shown in the figure, the data for the three driving styles also exhibit varied significant distributions in the feature space.

## 4. Personalized Decision-Making Model Based on LSTM

This section presents the modeling of the LC interaction behavior of vehicles, the fundamental model of LSTM, and the personalized LCD model. Recent breakthroughs in deep learning have made it feasible to extract high-level characteristics from raw data [24]. In this paper, the capacity of LSTM networks to capture complex characteristics in LC scenes is exploited to model LCD behavior on the basis of temporal feature sequences.

### 4.1. Modeling of LC Interaction Behavior of Vehicles

#### 4.1.1. Scenario Description of Vehicle LC Behavior

**Figure 3:**
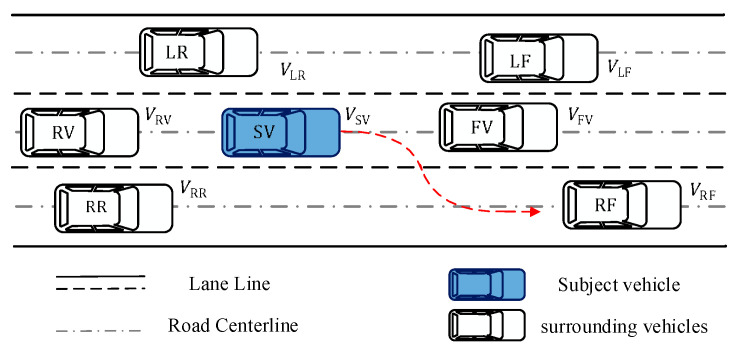
Illustration of lane change driving scenario.

Vehicle LC behaviors are typical and crucial activities performed by drivers in response to their driving demands, driving environment conditions, and traffic flow. To describe the LC behavior in a complicated environment, the LC driving scenario represented in Figure 3, below, is adopted. The surrounding vehicles are dispersed according to their location relative to the SV. Special emphasis placed on the fact that the SV is fitted with the decision system and accompanying sensors described in this paper, which are capable of capturing information about the surrounding vehicles in real time.

Depending the features of LC behavior and the research aims, the LC process is frequently divided into two stages [16]. The first stage is the LCD stage, which is the process of driver intention creation and route selection. The second stage is the LC execution stage, which consists of the identification of collision−free pathways and the tracking of the produced paths. However, this research only addresses the first stage. The precise meanings of the parameters in Figure 3 are presented in Table 3.

#### 4.1.2. LC Interaction Behavior Modeling

On the basis of the interaction information, the surrounding vehicles in real traffic scenarios are regarded as an interdependent whole, and their maneuvering behaviors affect each other’s decisions. To explain this interaction, the lane change benefit function described in [25] is borrowed, and the effects of SV travel efficiency, collision risk and driver comfort are considered. According to the literature [26], the behavioral decision making of a normal rational driver is a process of continuously pursuing the maximization of benefits.

The vehicle should be driven at as high a speed as possible under safe driving conditions, and the gain function of travel efficiency is set as shown in Equation (5), in which $V_{S,t}$ is the current speed of the SV, $V_{l}$ is the current speed limit of the road section, where $V_{l}=80\;\mathrm{Km}/h$ is taken, and $V_{f}$ is the current traffic speed of the road section.

(5) $$U_{\mathrm{efficiency}}=\left(V_{H,t}-V_{f}\right)/\left(V_{l}-V_{f}\right)$$

For the calculation of the collision risk benefit, it is necessary to first predict the trajectory of the SV and the surrounding vehicles during the future period T, and then judge the collision risk according to the trajectory.

- For the surrounding vehicles, use the constant turn rate and acceleration model (Constant Turn Rate and Acceleration, CTRA) to predict its trajectory during the future period T.
- For the SV, trajectory prediction needs to be performed according to its driving intention. If it is lane keeping, use the CTRA model to obtain its trajectory; if it is LC, use the quintic polynomial spline curve to fit its LC trajectory.

To improve the accuracy of the collision gain, here, the collision safety condition is defined. The collision benefit is characterized by the distance between vehicles, calculated on the basis of the collision safety condition. Assuming that the length and width of the SV and the surrounding vehicles $V_{q}$ are $L_{0},\;D_{0},\;L_{q},\;\mathrm{and}\;D_{q}$, respectively, the coordinates and heading angles of the two vehicles at a certain time t are ($x_{0}^{t},y_{0}^{t},\varphi_{0}^{t}$) and $\left(x_{q}^{t},x_{q}^{t},\varphi_{q}^{t}\right)$, and the difference between the heading angles of the two vehicles $\Delta \varphi =\varphi_{0}^{t}-\varphi_{q}^{t}$. Then, the conditions under which the *SV* and surrounding vehicles will not collide at any time t during the prediction period are:

(6) $$\left|\left(x_{0}^{t}-x_{q}^{t}\right)cos\varphi_{q}^{t}+\left(y_{0}^{t}-y_{q}^{t}\right)sin\varphi_{q}^{t}\right|\geq \frac{\sqrt{L_{0}+D_{0}}}{2}\sin\left(\alpha +\left|\Delta \varphi \right|\right)+\frac{L_{q}}{2}+\Delta s$$

Then, the gain function of the collision risk between the *SV* and the surrounding vehicle $V_{q}$ is:

(7) $$U_{SV,V_{q}}\;=\;\{\begin{array}{c} 0\;,\;\mathrm{match}\;\mathrm{safety}\;\mathrm{conditions}\\ -\frac{1}{d_{min}^{SV,V_{q}}},\;\;\mathrm{dismatch}\;\mathrm{safety}\;\mathrm{conditions} \end{array}\;$$

In the formula, $d_{min}^{SV,V_{q}}$ is the minimum distance between the SV and the surrounding vehicles $V_{q}$(qϵ{1,2,3,…,m}) during the prediction period T. The collision gain of the SV and all surrounding vehicles in the forecast period is as follows:

(8) $$U_{\mathrm{collision}}=\underset{t\in T,t\neq 0}{\sum }U_{SV,V_{q}}$$

During driving, drivers prefer to drive at a smooth speed; sharp acceleration, deceleration and frequent LC can affect their comfort. Here, the negative value of the sum of the squares of lateral acceleration $a_{x}$ and longitudinal acceleration $a_{y}$ integrated over the time period T is used as the comfort gain. The comfort gain can then be expressed as follows:

(9) $$U_{comfort}=-\int_{0}^{T}\left(a_{x}^{2}+a_{y}^{2}\right)dt$$

The travel efficiency gain $U_{efficiency}$, the collision gain $U_{\mathrm{collision}}$, and the comfort gain $U_{comfort}$ of the SV can be calculated using Equations (5), (8) and (9), above; then, the total gain is

(10) $$U_{total}=\omega_{1}U_{efficiency}+\omega_{2}U_{\mathrm{collision}}+\omega_{3}U_{comfort}$$

In the formula, $\omega_{1},\;\omega_{2}\;\mathrm{and}\;\omega_{3}$ are the weight coefficients of each gain. Using the NGSIM dataset, after normalizing the three returns, according to [26], the conjugate gradient method can be used to estimate the respective weight coefficients, as follows: $\omega_{1}$ = 0.2834, $\omega_{2}$ = 0.6426, $\omega_{3}$ = 0.3182.

### 4.2. The Basic LSTM Model

**Figure 4:**
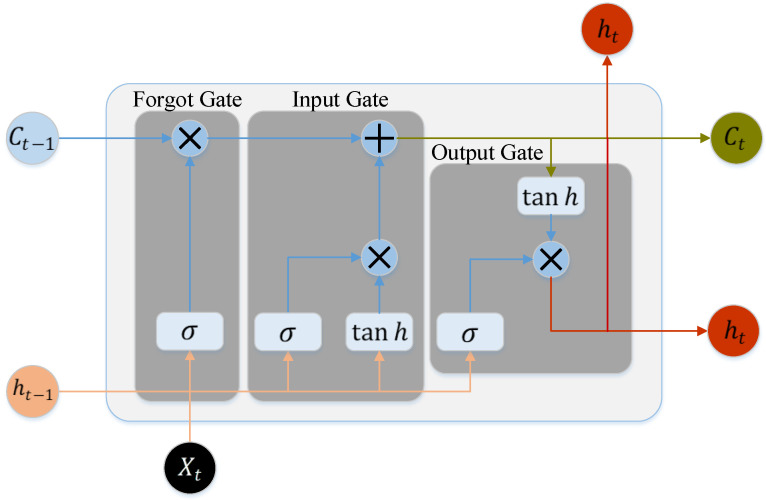
The LSTM cell structure.

The Long Short-Term Memory Neural Network (LSTM) is a recurrent neural network (RNN) structure [27]. LSTM overcomes the issue of gradient explosion and gradient disappearance present in RNN [28,29]. Unlike RNN, LSTM adds three additional ‘gate’ control structures, namely the forget gate, input gate and output gate. The storage unit is used to implement timing storage and timing prediction. The primary unit structure of LSTM is represented in Figure 4.

In the following, the LSTM cell structure and its concepts are described in further detail. Among these, the sigmoid function and the tanh function are two significant activation functions in the LSTM network structure. Their expressions are as follows.

(11) $$sigmoid\left(x\right)=\frac{1}{1+e^{-x}}$$

(12) $$\tanh\left(x\right)=\frac{e^{x}-e^{-x}}{e^{x}+e^{-x}}$$

(1) Forget gate: During the training period, the forget gate decides which information is to be abandoned. $f_{t}$ stands for forget gate, which is defined as follows:

(13) $$f_{t}=\sigma \left(W_{f}\left[h_{t-1},X_{t}\right]+b_{f}\right)$$

where $h_{t-1}$ is the output memory state of the LSTM in the previous cycle, and $X_{t}$ represents the state of the input.

(2) Input gate: The input gate determines which fresh information will be input. Its expression is as follows:

(14) $$i_{t}=\sigma \left(W_{i}\left[h_{t-1},X_{t}\right]+b_{i}\right)$$

(15) $${\tilde{C}}_{t}=tanh\left(W_{c}\left[h_{t-1},X_{t}\right]+b_{c}\right)$$

(16) $$C_{t}=f_{t}*C_{t-1}+i_{t}*{\tilde{C}}_{t}$$

In the expression for the input gate, $i_{t}$ represents the input gate, $\;{\tilde{C}}_{t}$ is the estimate for the new cell, and $C_{t}$ is the new long-term memory formed by this network.

(3) Output gate: The output gate regulates the output of the LSTM. Its expression is as follows:

(17) $$O_{t}=\sigma \left(W_{0}\left[h_{t-1},X_{t}\right]+b_{0}\right)$$

(18) $$h_{t}=O_{t}*tanh\left(C_{t}\right)$$

where $O_{t}$ represents the output of the network at this moment, and $h_{t}$ is the output of the new hidden state information. $W_{*}=\left(W_{f},W_{i},W_{c},W_{0}\right)$ represents the weight matrix of LSTM from unit to gate, and $b_{*}=\left(b_{f},b_{i},b_{c},b_{0}\right)$ represents the bias vector of each gate.

### 4.3. Building the Personalized LCD Model

In this paper, LSTM is mainly used to process the LC feature vector and LC gain data of the SV’s personalized and generate driving decision instructions. LSTM is able to obtain driving features at different timestamps, and then mine higher−level time series features to represent driving memory effects [30]. Due to the incorporation of historical driving information, the LSTM model is able to achieve improved predictions.

In actual traffic scenarios, the driver’s LC conduct is impacted by several factors. In addition to the microscopic driving state of the driver themselves and the surrounding vehicles, drivers have to consider other factors in order to make the best LCD [31]. On the basis of the experience of previous researchers [32,33] regarding the selection of LCD feature parameters, 20 LCD feature parameters were selected and added to the LC gain values in this paper, as shown in Table 4. It is important to note, in particular, that the features used for the LCD are different from those used for the clustering of driving styles in Table 1. Since LC is a continuous process, its requirements need to be calculated at each time step. Driving style, on the other hand, is constant throughout a particular time period, and therefore does not need to be measured as regularly.

**Figure 5:**
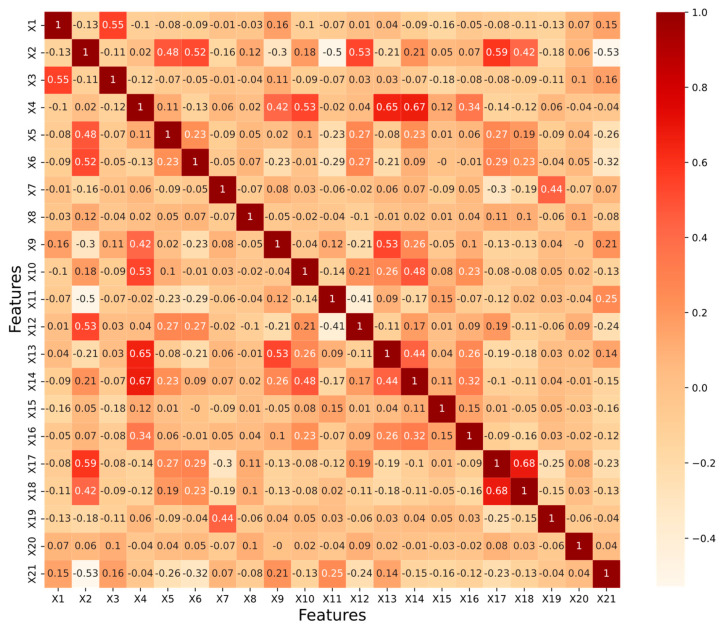
Correlation matrix among features.

The feature parameters of the vehicle are directly transferred to vectors as model input, which may lead to data redundancy. To check whether there is redundancy of information between features, the correlation of feature parameters was analyzed using Spearman’s coefficients [10]. The correlation matrix between the features is shown in Figure 5, where the values are the correlation coefficients m, and m∈[−1, 1]. The closer the value of m is to ±1, the higher the linear correlation between the random variables $x_{i}$ and $x_{j}$. Usually, if |m|>0.5, a significant correlation between the variables is implied. Similarly, the deeper the color in the correlation matrix, the higher the connection between the two traits, suggesting that they contain more related information. To filter out redundant variables, *m* = 0.6 was selected as the threshold value. If a pair of variables has a correlation coefficient *m* ≥ 0.6, one of them will be deleted to speed up model training.

**Figure 6:**
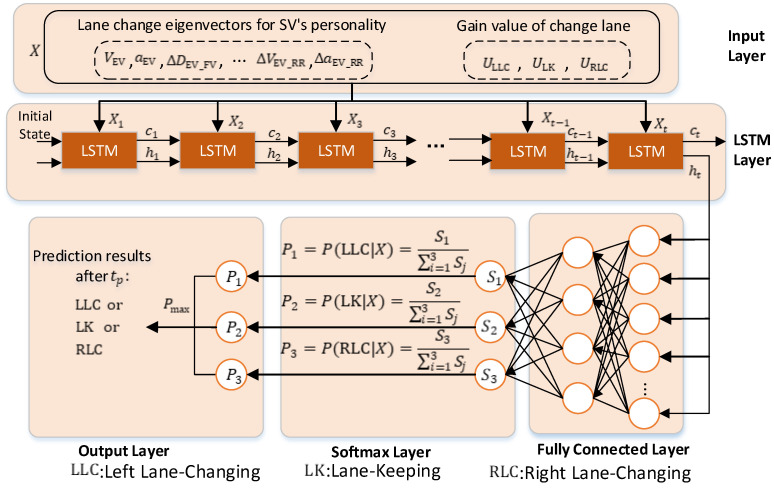
Structure of the lane change decision model.

To achieve a personalized LCD, in this paper, a decision model is developed, as shown in Figure 6. The structure of the model contains five layers, where the input layer is used to receive information on the state of the SV and the surrounding vehicles. The data accepted by the input layer are time series variables that characterize the lane changing behavior of the SV, the length of which is denoted by $t_{p}$, and the data for each time step are as follows:

(19) $$X=\left[X_{1}^{t},X_{2}^{t},X_{3}^{t},\ldots ,U_{LLC}^{t},U_{LK}^{t},U_{RLC}^{t}\right]$$

where $\left[X_{1}^{t},X_{2}^{t},X_{3}^{t},\ldots \;X_{20}^{t}\right]$ is the decision feature vector extracted from the SV trajectory with known driving style, and $U_{LLC}^{t},U_{LK}^{t}$ and $U_{RLC}^{t}$ are the gain values of the SV performing left LC, lane keeping and right LC at time t, respectively.

The second layer of the model’s structure is composed of a two−layer LSTM neural network. This LSTM network layer captures the key factors influencing vehicle behavior decisions from the high−dimensional time series data and then classifies the input data appropriately. The third layer is a fully connected layer, which is used to transform the dimensions of the transmitted data. The fourth layer is the Softmax layer, which is processed by the Softmax classifier to obtain the LC behavior probability matrix of the SV. This probability matrix is as follows:

(20) $$P=\left[P_{1},P_{2},P_{3}\right]$$

where $P_{1},P_{2}$ and $P_{3}$ are the probabilities of left LC, lane keeping and right LC for the SV, respectively. The fifth layer is the output layer of the model, which obtains the lane change decision results at the prediction time $t_{p}$. According to [34], the LC time in a highway environment is generally between 3.5 s and 6 s, and a complete LC process can be performed in an average of 5 s. However, this paper only considers the LC decision, so $t_{p}=3\;s$ is chosen.

The aim of this LCD model is to enable the SV to generate safe, stable, and personalized LC decisions on the basis of its own driving state and the location information of surrounding vehicles. Firstly, the LC feature parameters of the SV are extracted on the basis of GMM clustering. Then, the personalized LC feature parameters and LC gain values are utilized as feature inputs to train the model and generate the resulting LCD.

## 5. Model Evaluation and Analysis

In this section, the LCD model is evaluated using multiple evaluation metrics, and a comparison with other models is performed. Furthermore, LCD models considering various driving styles are evaluated. The training tasks for the dataset pre−processing and decision models in this paper were performed by MATLAB 2020a and pytorch-based jupyter on a laptop in the Windows 10 environment.

### 5.1. Data Preparation

In this paper, data were extracted from the NGSIM dataset to train the decision model. On the basis of the recognition of SV driving style, further LC decision parameters were extracted. Sequential data with a predicted time length of 3 s between the SV and surrounding vehicles were extracted using the sliding window method. At the same time, the personalized LCD samples were labeled with data. The left LC, lane keeping, and right LC were represented using 0, 1 and 2, respectively. Then, 80% of the cases were randomly selected from the total sample data as training samples for the decision model, while the remaining 20% were used as test samples.

### 5.2. Model Parameter Settings

In this paper, the LSTM network is used to learn the interaction characteristics of the vehicle, and the behavioral decision of the SV is output through Softmax. The parameter settings for the LSTM network model are shown in Table 5. To achieve better model training accuracy, we set the batch size to 512, the number of nodes in the hidden layer to 256, and the number of iterations for model training to 300. The cross−entropy function is used to train the LSTM network model. Importantly, a learning rate that is too high will cause the loss function to ignore the minimum loss, while a learning rate that is too small will make training exceedingly sluggish. On the basis of previous research and experience [35], the optimal learning rate was finally determined to be 0.0124 by plotting the learning rate against training loss.

### 5.3. Results and Analysis

#### 5.3.1. Evaluation Indicators of the Model

The model is trained using the training set, and the model performance is evaluated using the test set. Performance metric testing involves the following.

(1) Accuracy rate $A_{cc}$: Indicates the proportion of the number of correct predictions in the model test instance to the total number of test instances, and its calculation formula is as follows:

(21) $$A_{cc}=\frac{T_{p}+T_{N}}{T_{p}+T_{N}+F_{P}+F_{N}}$$

where $T_{p}$ is the number of samples where the true label is positive and the prediction is also positive; $T_{N}$ is the number of samples where the true label is negative and the prediction is also negative; $F_{P}$ is the number of samples where the true label is negative and the prediction is positive; and $F_{N}$ is the number of samples where the true label is positive and the prediction is negative.

(2) $F_{1}$ score: Refers to the harmonic mean of the precision rate *P* and the recall rate *R*, and its calculation formula is as follows:

(22) $$P=\frac{T_{P}}{T_{p}+F_{p}}$$

(23) $$R=\frac{T_{P}}{T_{p}+F_{N}}$$

(24) $$\;F_{1}=\frac{2*P*R}{P+R}$$

(3) Test loss $T_{loss}$: The cross−entropy loss of the decision model on the test set; its expression is as follows:

(25) $$T_{loss}=-\frac{1}{m}\overset{m}{\underset{i=1}{\sum }}\overset{N}{\underset{n=1}{\sum }}P_{iN}\log\left(q_{iN}\right)$$

where *m* is the number of samples tested by the model, *n* is the LC decision intention, $P_{iN}$ is the true label of the ith sample in the test set belonging to the Nth decision intention, and $q_{iN}$ is the probability of the model predicting that the *i*th sample belongs to the Nth decision intention.

In the NGSIM dataset, the number of cars executing lane-maintaining actions is substantially larger than the number of cars performing LC. From the previous LC data extraction, it can be recognized that the number of right LC is smaller than the number of left LC. When the multi−class data are uneven, the accuracy evaluation method is flawed. Therefore, this study presents assessment measures such as accuracy rate, recall rate, and F1 score. Meanwhile, the Macro-average receiver operating characteristic curve (ROC curve) and the area under the curve (Macro-AUC) are also utilized to assess the performance of the LCD model.

#### 5.3.2. Decision Model Evaluation and Comparison

To verify the effectiveness of the LCD method proposed in this paper, the relevant performance metrics of several models are compared and analyzed using the same dataset. Here, the LCD model LSTM_I proposed in this paper is compared with five other algorithmic models. These are: the LSTM model, the SVM model [11], the Logistic Regression (LR) model [36], the KNN model [37] and the XGB model [10]. Of these, the LSTM model is a single LCD model that does not take into consideration the introduction of interaction feature parameters. In addition, the other models (SVM, LR, KNN and XGB) are applied using Python and the “scikit-learn” package. It should be noted that this part of the dataset does not specify the driving style of each vehicle.

The optimization of model parameters is an issue that needs to be considered here. Common model parameter optimization methods include genetic algorithm, random search, and Bayesian optimization. Compared with other optimization algorithms, the Bayesian approach exhibits superior performance in fewer iterations [38]. Therefore, the Bayesian method is selected here to optimize the model. A selection of critical parameters and variables following the optimization of each model is shown in Table 6.

**Figure 7:**
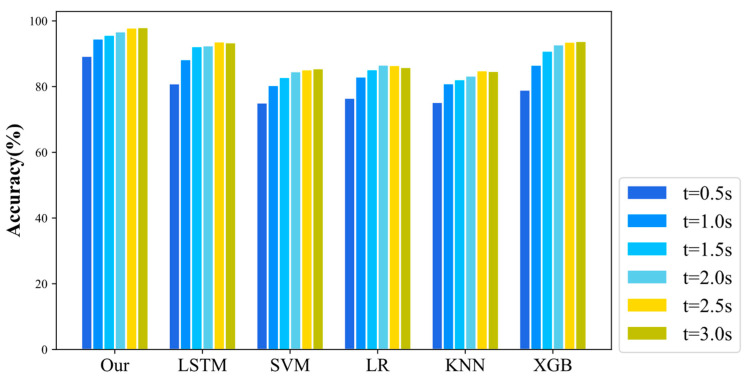
Accuracy at different time points.

In this paper, the performance of the six decision models is tested using data under different time windows. To improve the real-time performance and accuracy of the decision models, the upper limit of the time length was chosen to be 3 s. The accuracy of the different LCD models was calculated for time windows of 0.5, 1.0, 1.5, 2.0, 2.5, and 3 s, respectively, as shown in Figure 7. As can be seen from Figure 7, the overall accuracy of the model increases as the dataset increases. The accuracy of all models stabilized when the data time length reached 2.5 s. Furthermore, the accuracy of our models already exceeded 95% at a time length of 2.0 s for the test data. Compared with the LSTM model without the introduction of interactions, our model demonstrated an improvement in overall accuracy by 4.26%. Compared with SVM, LR, KNN and XGB, the overall accuracy was improved by 8.89%, 11.04%, 14.65% and 4.96%, respectively. This shows that the introduction of the interaction module did improve the accuracy of the autonomous driving LCD.

The LCD model is established 3 s before LC. The six LC models are trained and tested on the basis of datasets I−80 and US−101, and the results are shown in Table 7. As can be seen from Table 7, the LSTM_I model has combined accuracy, recall, and F1 score values of 0.965, 0.951 and 0.956, respectively, demonstrating better classification accuracy than the other models. This indicates that the model is better able to mine the potential features in the LC process and generate more accurate decision results. Additionally, the test loss values of different decision models are presented in Table 7. From Table 7, it can be seen that our model also shows better performance in terms of test loss values. This further demonstrates that the decision model is capable of making more accurate predictions.

**Figure 8:**
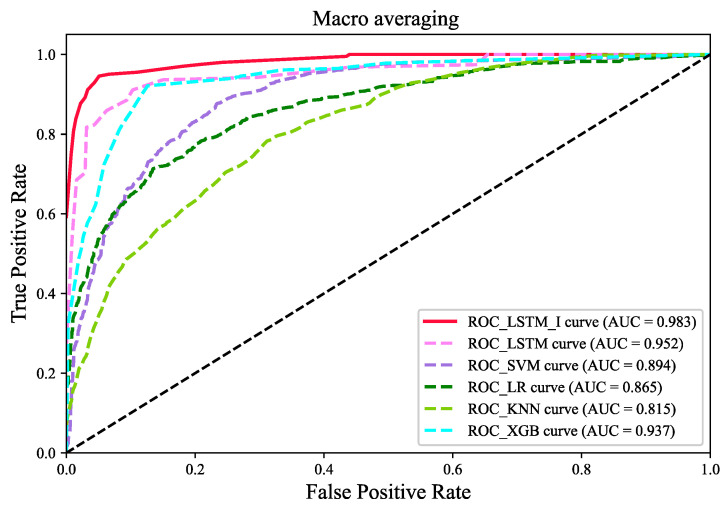
The Macro-average ROC curve of each model.

Figure 8 shows the macroscopic receiver operating characteristic curves of the six models. Among them, the KNN algorithm model has the worst performance, with a Macro−AUC value of 0.815. The Macro−AUC value of the SVM model is slightly higher than that of the logistic regression model, with Macro−AUC values of 0.894 and 0.865, respectively. In addition, the LSTM model algorithm exhibits better performance than the XGB model algorithm, with Macro−AUC values of 0.952 and 0.937, respectively. Meanwhile, the LCD model LSTM_I proposed in this paper shows the best performance, with a Macro−AUC value of 0.983.

In general, the LCD model proposed in this paper shows better performance than the other decision models. The reason for this is that, during the process of traffic flow, the SV and the surrounding vehicles form an interacting whole. The behavioral decisions of the SV are not only influenced by its own state, but also by the driving environment and the behavior of the surrounding vehicles. In addition, the LCD model proposed in this paper is able to learn the correlation between vehicles on the basis of the interaction and mine the key features affecting LC. Under the same driving environment, this decision model is able to make more reasonable behavioral decisions on the basis of the surrounding vehicle information and its own state.

#### 5.3.3. Model Evaluation Considering Driving Style

In this section, the LSTM_I model is further evaluated using the various driving style data obtained in Section 3. The classification results of the test data for different driving styles are shown in Table 8. From Table 8, it can be seen that the decision model has high accuracy in predicting non-LC behavior, while it has the lowest accuracy in predicting right LC behavior. Meanwhile, among the different driving styles, moderate has the highest overall accuracy, followed by conservative and aggressive, from high to low, respectively. This is because there is an imbalance in the data of the three driving styles extracted in this paper, and more feature data could improve the accuracy of the model to a certain extent. In addition, the prediction accuracy when taking different driving styles into consideration was overall higher than that for samples without driving style classification.

Table 9 shows the prediction accuracy of the six models for predictions based on data related to driving behavioral characteristics for different driving styles. The data in the table are the average of the prediction accuracy of each model for behaviors such as LCL, LK and RCL. From Table 9, it can be seen that the overall accuracy of the LSTM_I model is better than that of the other five models, both for classified and non-classified samples. Among them, the average accuracy of the LSTM_I model prediction, performed on the basis of three driving style feature data points, is 97.28%, while the accuracies of the other five models are 92.98%, 86.29%, 84.32%, 91.69% and 81.35%, respectively. In addition, the same conclusion as that presented in Table 8 can also be obtained: for the six LCD models, the prediction accuracy following classification is higher than that for the unclassified samples.

**Figure 9:**
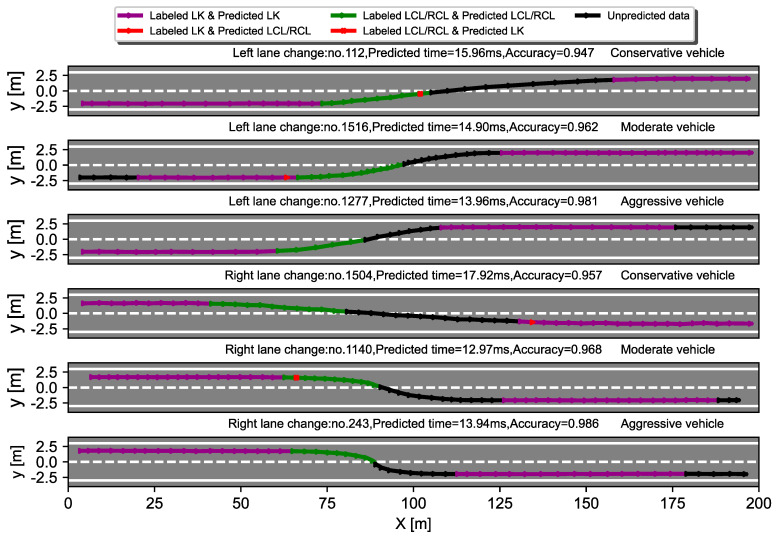
Examples of lane change prediction for vehicles with different driving styles. Purple and green represent correctly predicted data segments for LC and LK, respectively, black represents unpredicted data, and red represents incorrectly predicted data.

Figure 9 shows several examples of lane change predictions for vehicles with different driving styles. Here, we use the LSTM_I model to predict the driving behavior of real vehicles found in the NGSIM data, and we color-code the actual trajectories of the predicted vehicles according to the model’s predictions. In this case, the data segments that correctly predict the LK and LC behavior are represented in purple and green, respectively. Data segments that are not involved in the prediction are marked in black, while data that predict LK and LC incorrectly are shown in red. As can be seen from Figure 9, the model has an overall prediction accuracy of over 94% for vehicles with different driving styles. In contrast to Table 8, the prediction accuracy is higher for aggressive driver behavior. In addition, the model has a prediction time range of 12 ms to 18 ms, which is sufficient for real-world situations. Overall, the decision model proposed in this paper is able to produce good lane change predictions for drivers with different driving styles.

## 6. Conclusions

This research proposes an LCD model that considers SV driving styles as well as interactions. Feature variables such as speed, acceleration and headway time distance are selected, and the style type is identified for each vehicle sample on the basis of the unsupervised clustering algorithm GMM. The interaction between the SV and the surrounding vehicles is described by constructing a gain function, which takes into account the safety, driving efficiency, and comfort of the SV. The LC feature variables with different driving styles and the LC gain values are used as model inputs to construct an LSTM−based personalized LCD model.

To verify the effectiveness of the model, real vehicle trajectory data, MGSIM, were used to evaluate the model. The model was also compared with other models, and the results showed that the model outperformed the other models in terms of accuracy, F1 score, and macro−AUC value. This indicates that the model is able to make more accurate behavioral decisions on the basis of the state of its own vehicle and information regarding the surrounding vehicles. Behavioral decisions were also evaluated for different driving styles. The test results showed that the personalized LCD framework is able to make sound decisions on the basis of different driving styles, and it can provide personalized options for different drivers.

Future work will focus on the improvement of the proposed model algorithms and the application of real−time on−board hardware systems for algorithm validation. Additionally, other traffic participants (e.g., pedestrians, non-motorized vehicles) will be taken into consideration in the driving environment in order to further improve the decision−making capability of autonomous vehicles.

## Figures and Tables

**Table 1:** Statistical description of the features used for driving style recognition.

Symbol	Features	Statistic Values		
ACC	Acceleration	Max Acceleration	Mean Acceleration	Acceleration STD
TH	Time Headway	Max Time Headway	Mean Time Headway	Time Headway STD
Jerk	Jerk	Max Jerk	Mean Jerk	Jerk STD
VX	Lateral speed	Max Lateral speed	Mean Lateral speed	Lateral speed STD
VY	Longitudinal speed	Mean Longitudinal speed	Longitudinal speed STD	
SH	Space Headway	Mean Space Headway	Space Headway STD	

**Table 2:** Comparison of characteristic parameters of drivers with different driving styles.

**Styles**	**VY Mean**	**VY STD**	**VX Max**	**VX mean**	**VX STD**	**ACC Max**	**ACC Mean**	**ACC STD**
Conservative	9.391	3.305	1.260	0.595	0.406	3.122	2.670	0.519
Moderate	11.310	2.426	1.337	0.618	0.316	3.715	3.257	0.443
Aggressive	12.682	1.590	1.568	0.721	0.397	4.842	4.006	0.547
**Styles**	**TH Max**	**TH Mean**	**TH STD**	**Jerk Max**	**Jerk Mean**	**Jerk STD**	**SH Mean**	**SH STD**
Conservative	0.864	0.597	0.674	2.936	−0.056	1.025	34.240	4.764
Moderate	0.565	0.431	0.457	2.518	−0.073	1.106	28.973	3.787
Aggressive	0.540	0.276	0.982	2.637	−0.086	0.901	23.651	1.368

**Table 3:** The meanings of parameters in the lane-changing scene.

SV	The Subject Vehicle
LF /V/RF	The vehicle in front of SV in left/middle/right lane
LR/RV/RR	The vehicle in front of SV in left/middle/right lane
$V_{LF}$ $/V_{FV}/V_{RF}$	The speed of vehicle in front of SV in left/middle/right lane
$V_{LR}$ $/V_{RV}/V_{RR}$	The speed of vehicle in behind of SV in left/middle/right lane

**Table 4:** Statistical description of characteristic parameters of driving decisions.

Number	Features	Description
$X_{1}$	$V_{SV}$	Speed of the SV
$X_{2}$	$a_{SV}$	Acceleration of the SV
$X_{3}$	$\Delta D_{SV\_FV}$	The distance between SV and FV
$X_{4}$	$\Delta V_{SV\_FV}$	The speed difference between SV and FV
$X_{5}$	$\Delta a_{SV\_FV}$	The acceleration difference between SV and FV
$X_{6}$	$\Delta D_{SV\_RV}$	The distance between SV and RR
$X_{7}$	$\Delta V_{SV\_RV}$	The speed difference between SV and RR
$X_{8}$	$\Delta a_{SV\_RV}$	The acceleration difference between SV and RR
$X_{9}$	$\Delta D_{SV\_LF}$	The distance between SV and LF
$X_{10}$	$\Delta V_{SV\_LF}$	The speed difference between SV and LF
$X_{11}$	$\Delta a_{SV\_LF}$	The acceleration difference between SV and LF
$X_{12}$	$\Delta D_{SV\_LR}$	The distance between SV and LR
$X_{13}$	$\Delta V_{SV\_LR}$	The speed difference between SV and LR
$X_{14}$	$\Delta a_{SV\_LR}$	The acceleration difference between SV and LR
$X_{15}$	$\Delta D_{SV\_RF}$	The distance between SV and RF
$X_{16}$	$\Delta V_{SV\_RF}$	The speed difference between SV and RF
$X_{17}$	$\Delta a_{SV\_RF}$	The acceleration difference between SV and RF
$X_{18}$	$\Delta D_{SV\_RR}$	The distance between SV and RR
$X_{19}$	$\Delta V_{SV\_RR}$	The speed difference between SV and RR
$X_{20}$	$\Delta a_{SV\_RR}$	The acceleration difference between SV and RR
$X_{21}$	$U_{total}$	The total lane change profit value

**Table 5:** The settings of the LSTM network model.

Item	Description	Value
Batch size	Batches per training	512
Hidden size	Number of hidden neural units of LSTM	256
Epoch	The number of iterations for LSTM model training	300
Output size	LSTM model output size	3
Learning rate	Learning rate	0.0124
Loss function	Instruct LSTM to update weight parameters	CrossEntropy

**Table 6:** Key parameters following the optimization of each model.

Model	Optimal Parameter
SVM	C = 7.42; gamma = 0.001; kernel: ‘rbf’
LR	Penalty: ‘l2’; tol = 1e − 4; max_iter = 640; solver: ‘sag’; multi_class = ‘multinomial’
XGB	learning_rate = 0.0124; max_depth = 6; min_child_weight = 0.1; gamma = 0.36
KNN	n_neighbors = 5; leaf_size = 30, weights: ‘uniform’; algorithm = ‘auto’

**Table 7:** Performance comparison of the different approaches.

Model	Precision (%)			Recall (%)			F1-score (%)			*T_loss_*
	LCL	LK	LCR	LCL	LK	LCR	LCL	LK	LCR	
LSTM_I	96.54	97.33	95.46	98.24	96.57	90.37	97.28	97.10	92.84	0.127
LSTM	94.42	92.26	91.51	88.04	94.01	89.70	93.67	93.19	92.60	0.210
SVM	89.03	86.49	88.43	84.18	90.07	80.59	87.08	88.44	83.23	0.267
LR	86.21	90.54	80.60	84.36	83.28	83.16	84.50	85.39	81.62	0.368
XGB	93.86	91.47	90.33	95.06	92.39	81.64	94.16	91.22	85.35	0.156
KNN	76.63	81.68	84.20	80.17	82.54	83.66	78.84	81.40	83.59	0.423

**Table 8:** Prediction accuracy of LCD model considering driving style.

Decision Results	Driving Style		
	Left Lane Changing	Lane Keeping	Right Lane Changing
Conservative	96.95%	98.87%	94.57%
Moderate	97.94%	99.79%	96.59%
Aggressive	94.85%	97.68%	92.29%
Mean	96.58%	98.78%	94.48%
Non-Classified	94.18%	98.14%	92.10%

**Table 9:** The prediction accuracy of 6 models considering the driving style of the SV.

Driving styles	LSTM_I	LSTM	SVM	LR	XGB	KNN
Conservative	96.80%	91.76%	84.61%	85.65%	90.58%	81.94%
Moderate	98.46%	95.31%	92.62%	90.03%	95.31%	86.43%
Aggressive	96.57%	91.89%	81.64%	77.27%	89.18%	74.67%
Mean	97.28%	92.98%	86.29%	84.32%	91.69%	81.35%
Non-Classified	96.51%	91.59%	83.96%	82.81%	89.89%	77.2%

## Data Availability

Not applicable.
